# Emotion regulation moderates the association between COVID-19 stress and mental distress: findings on buffering, exacerbation, and gender differences in a cross-sectional study from Norway

**DOI:** 10.3389/fpsyg.2023.1121986

**Published:** 2023-06-22

**Authors:** Annie Haver, Henning Krampe, Lars Johan Danbolt, Gry Stålsett, Tatjana Schnell

**Affiliations:** ^1^Faculty of Social Sciences, Norwegian School of Hotel Management, University of Stavanger, Stavanger, Norway; ^2^School of Psychology, Faculty of the Arts, Social Sciences, and Humanities, University of Wollongong, Wollongong, NSW, Australia; ^3^Department of Anesthesiology and Intensive Care Medicine, Charité - Universitätsmedizin Berlin, Corporate Member of Freie Universität Berlin and Humboldt-Universität zu Berlin, Berlin, Germany; ^4^MF Norwegian School of Theology, Religion and Society, Majorstuen, Oslo, Norway; ^5^Centre for Psychology of Religion, Innlandet Hospital Trust, Brumunddal, Norway; ^6^Modum Bad Psychiatric Center, Vikersund, Norway; ^7^Existential Psychology Lab, Institute of Psychology, University of Innsbruck, Innsbruck, Tyrol, Austria

**Keywords:** COVID-19, anxiety, depression, emotion regulation, moderator analysis, public mental health, reappraisal, suppression

## Abstract

**Background:**

Maintaining good mental health is important during a crisis. However, little attention has been given to how people achieve this, or how they evaluate emotions associated with stressors, such as the COVID-19 pandemic. This study aims to (1) investigate whether emotion regulation, in particular cognitive reappraisal and suppression, moderates the relationship between COVID-19 stress and general mental distress and (2) examine gender differences in the interrelations between COVID-19 stress, emotion regulation, and mental distress.

**Methods:**

Data from a population in Norway (*n* = 1.225) were collected using a cross-sectional survey during the early months of the COVID-19 pandemic. Emotion regulation was measured using the Emotion Regulation Questionnaire Scale (ERQ), COVID-19 stress with the COVID-19 Stress Scale, and mental distress with the Patient Health Questionnaire 4 (PHQ-4). Moderation analyses were conducted using the PROCESS macro for SPSS.

**Results:**

There was a strong association between COVID-19 stress and general mental distress (*r* = 0.61). The moderation analyses showed substantial moderation effects of cognitive reappraisal and suppression on the relationship between COVID-19 stress and mental distress. Cognitive reappraisal served as a buffer (*p* = 0.001) and suppression (*p* = 0.002) exacerbated the relation between COVID-19 stress and mental distress. Men had higher scores of suppression (*p* < 0.001), and women had higher scores of cognitive reappraisal (*p* = 0.025). The buffering effect of cognitive reappraisal presented itself only in women (*p* < 0.001), while the exacerbation effect of suppression appeared only in men (*p* < 0.001).

**Conclusion:**

The current study suggests that COVID-19 pandemic-related stress is easier to deal with for those who have the tendency to cognitively reappraise. In contrast, suppression is associated with symptoms of depression and anxiety. The prevention of mental distress can be supported by guiding people about the importance of using healthy emotion regulation strategies, as well as helping them to become more aware of the way they interpret and regulate their emotions. Gender differences in emotion regulation suggest gender awareness, e.g., tailored programs for men and women.

## Introduction

In early 2020, SARS-CoV-2 infected millions of people and the death toll associated with COVID-19 increased rapidly worldwide (WHO, 2020; Schöley et al., 2022). To limit and delay the COVID-19 outbreak, societies went into lockdown, and both the pandemic and its aftermath changed the daily life of most people globally (OECD, 2021; WHO, 2022b). During the first year of the pandemic the global prevalence of anxiety and depression increased by 25% (WHO, 2022a). This increase in mental distress is documented in numerous systematic reviews and meta-analyses reporting that the COVID-19 pandemic has generated major psychological health problems worldwide (e.g., Cénat et al., 2021; Wu et al., 2021; Robinson et al., 2022). Today, research shows that many people have rebounded from the mental distress caused by the pandemic, mostly returning to previous health. However, a small but significant number of individuals are still suffering from mental health problems (Lopez-Leon et al., 2021; Saunders et al., 2021; Robinson et al., 2022). Moreover, mental health problems are associated with long COVID, a chronic condition with high incidence rates (Lopez-Leon et al., 2021; Sachs et al., 2022).

According to the Lancet COVID-19 commission, governments around the world were too slow to acknowledge the seriousness of the pandemic and act with urgency in response (Sachs et al., 2022). Norway with a population of 5.3 million people was probably among the first countries to implement a nationwide lockdown on March 12, 2020 (Ursin et al., 2020; Andreas, 2021). The lockdown in Norway included school closures, working from home and limitations on nonessential services (sports, non-essential business etc.). Overall, only grocery stores, pharmacies, and gas stations remained open. The authorities also implemented forced self-isolation for those at risk of infection and those who were infected. In addition, they implemented several quarantine restrictions, travel restrictions, prohibition from staying overnight in holiday cabins across municipal borders, as well as spatial distancing and the compulsory wearing of face masks in public places (Ursin et al., 2020; Andreas, 2021; Blix et al., 2021). Experiencing the outbreak of a novel and life-threatening virus disease, together with the restrictions on individuals’ autonomy and freedom, seems to have prompted an increase in mental distress in many people in Norway, particularly during the first months of the COVID-19 outbreak (Hoffart et al., 2020; Blix et al., 2021; Ebrahimi et al., 2021; Ernstsen and Havnen, 2021; Lassen et al., 2022). Even though Norway’s mortality and infection rate from Sars-CoV-2 were low compared to many other countries (Hvide and Johnsen, 2022), loneliness and fear were reported as substantial factors associated with mental distress during the outbreak (Hoffart et al., 2020; Bemanian et al., 2021; Blix et al., 2021; Ebrahimi et al., 2021).

Although individuals may face the same stressful event (for example COVID-19) there are individual differences in how associated negative emotions are experienced or regulated (Gross and John, 2003; Too and Butterworth, 2018; Raymond et al., 2019). Recent studies found that during the COVID-19 pandemic, several resources and resilience factors played moderating and/or mediating roles in the associations between stressful experiences and mental health, e.g., individuals’ sense of meaning of life (Schnell and Krampe, 2020), locus of control (Krampe et al., 2021), psychological flexibility (Smith et al., 2020), personality traits (Bacon and Corr, 2020; Smith et al., 2020; Liu et al., 2021; Abdelrahman, 2022; Lassen et al., 2022), as well as emotion regulation abilities (Xu et al., 2020; Liang et al., 2021; Ye et al., 2021; Gullo et al., 2022; Vertsberger et al., 2022). These findings imply that the way individuals deal with negative emotions in their daily lives, as well as under long term crises (e.g., pandemic), can either be a motivation for coping or it can have a negative impact on mental health and well-being (Garland et al., 2011; Conway et al., 2013; Xu et al., 2020).

Gross (1998b) differentiates between two emotion regulation strategies in his process model of emotion regulation: cognitive reappraisal and suppression. Both cognitive reappraisal and suppression behaviour operate along a continuum from conscious, effortful controlled regulation of emotions to unconscious, effortless, and automatic regulation (Gross and Thompson, 2006; Bargh and Williams, 2007). Emotion regulation is an important protective factor, and it concerns individuals’ attempts to control (modify) their emotions to respond in a flexible and adaptive way to the environment (Gross, 1998a; Boyes et al., 2016). Cognitive reappraisal involves a reinterpretation of a situation into a more positive light (Ford et al., 2017), changing its potential meaning. Notably, it is not the situation itself that is changed, it is the individual’s evaluation of the situation (Gross and Barrett, 2011; Raymond et al., 2019). Cognitive reappraisal can be employed prior to experiencing an emotion (Gross, 1998a), and it is therefore an effective strategy to reduce negative emotions through reframing emotion-eliciting experiences or stimuli (Gross and John, 2003). The second major emotion regulation strategy is suppression, involving the conscious inhibition of one’s emotion expressive behaviors, whether they are covert, overt or both (Lazarus and Alfert, 1964; Gross, 1998a). Suppression is a response-focused strategy that intervenes once an emotion is “under way,” or after the emotional response has been triggered (Gross, 1998a). For example, an individual may not be able to express their anxiety or fear about COVID-19, and may thus suppress their outer expression of negative emotion by putting on a façade of control, mutual agreement, or by becoming “paralyzed” by overwhelming negative emotions and therefore being unable to move forward. An individual inner emotion would thus remain unchanged, meaning that the expression of negative emotions is suppressed.

### Literature review

Cognitive reappraisal and suppression differ in their adaptiveness in regard to promoting or undermining psychological health (for review, see Aldao and Nolen-Hoeksema, 2010). Extensive research shows that cognitive reappraisal is beneficial for psychological health (for review, see Webb et al., 2012; Hu et al., 2014), and that it is linked to resilience, positive affect, mental well-being, increased life satisfaction, better job performance, as well as favorable cognitive and social outcomes (Gross and John, 2003; John and Gross, 2004; Kashdan et al., 2006; Kraiss et al., 2020). In contrast, suppression is considered a maladaptive strategy, associated with worse psychological health outcomes (Aldao et al., 2010; Hu et al., 2014; Chervonsky and Hunt, 2017; Cameron and Overall, 2018). The tendency to withhold the expression of emotions is linked to, for example, impaired interpersonal relationships, greater anxiety and depression, poorer life satisfaction, lack of authenticity, lower self-esteem and increased negative emotions (Gross and John, 2003; Kashdan et al., 2006; English and John, 2013). The differential effects of cognitive reappraisal and suppression have also been confirmed in several correlational studies during the pandemic (Cardi et al., 2021; Liang et al., 2021; Low et al., 2021; Santi et al., 2021; Ye et al., 2021; Gullo et al., 2022).

Moreover, previous research found that emotion regulation is a significant moderator of the association between stress and symptoms of anxiety and depression. While cognitive reappraisal seems to buffer the relationship between stress and symptoms of anxiety and depression (Troy et al., 2010; Vanderhasselt et al., 2014; Johnson et al., 2016), suppression seems to exacerbate the relationship between stress and anxiety/depression (Boyes et al., 2016; Hosogoshi et al., 2020). Recent COVID-19 studies also investigated the moderating role of emotion regulation in diverse populations. The majority of the investigations found that cognitive reappraisal buffered the positive relationships between diverse types of perceived stress and subjective health (Prikhidko et al., 2020; Xu et al., 2020; Yang et al., 2020; Gröndal et al., 2021; Kuhlman et al., 2021; Raio et al., 2021; Ye et al., 2021; Chen et al., 2022; Vertsberger et al., 2022), while suppression exacerbated these relationships (Wu et al., 2021; Ye et al., 2021; Chen et al., 2022). However, some investigations did not find the buffering effect of cognitive reappraisal (Zhang et al., 2021) or the exacerbating effect of suppression (Yang et al., 2020; Gröndal et al., 2021; Raio et al., 2021).

Various studies indicate gender differences in emotion regulation (Tamres et al., 2002; Nolen-Hoeksema, 2012; Rogier et al., 2019). Women generally seem to use more emotion regulation strategies than men, including cognitive reappraisal, but not expressive suppression (Tamres et al., 2002; Goubet and Chrysikou, 2019). Importantly, data suggest that women apply emotion regulation strategies in more flexible ways (Goubet and Chrysikou, 2019). Concerning gender differences in the use of the specific strategies of cognitive reappraisal and suppression, the evidence is inconsistent. Several researchers showed that women have a stronger tendency to use cognitive reappraisal (Tamres et al., 2002; Nolen-Hoeksema and Aldao, 2011; Megıas-Robles et al., 2019; Rogier et al., 2019), while men seem to tend toward expressive suppression (Flynn et al., 2010; Megıas-Robles et al., 2019; Rogier et al., 2019; Santi et al., 2021). However, some studies did not find these differences for either cognitive reappraisal (Santi et al., 2021) or suppression (Tamres et al., 2002; Nolen-Hoeksema and Aldao, 2011). The evidence is also inconclusive regarding the interplay of emotion regulation, stress and mental distress. On the one hand, convincing data suggest that the interrelations of emotion regulation and forms of stress and mental distress are rather similar in women and men (Aldao and Nolen-Hoeksema, 2012; Lutz et al., 2022). On the other hand, a variety of gender differences have been reported. For example, in several studies, gender moderated the associations of reappraisal and suppression with diverse mental health outcomes, with significant effects sometimes only in men (Flynn et al., 2010; Rogier et al., 2019; Jiang et al., 2022), sometimes only in women (Nolen-Hoeksema, 2012; Rogier et al., 2019), and sometimes in different ways in men and women (Flynn et al., 2010; Megıas-Robles et al., 2019; Zhang et al., 2020; Jiang et al., 2022). Gender analyses of emotion regulation are sparse in psychosocial COVID-19 research. While Li et al. (2022) found higher reappraisal scores in women and higher suppression scores in men, Canlı and Karaşar (2020) and Santi et al. (2021) reported higher suppression scores in men, but no gender differences for reappraisal. Rodas et al. (2022) stated some moderation effects of gender but they did not describe these. Muñoz-Navarro et al. (2021) found that emotion regulation strategies mediated the associations between COVID-19 worries and anxiety differently in women and men. Finally, Panno et al. (2022) showed that cognitive reappraisal was negatively associated with COVID-19 stress and general mental distress in women but not in men, while there were no gender effects for the association of suppression with COVID-19 stress and mental distress. To our knowledge, until now, gender comparisons have not been published for the buffering and exacerbating effects of reappraisal and suppression, respectively.

**Figure 1:**
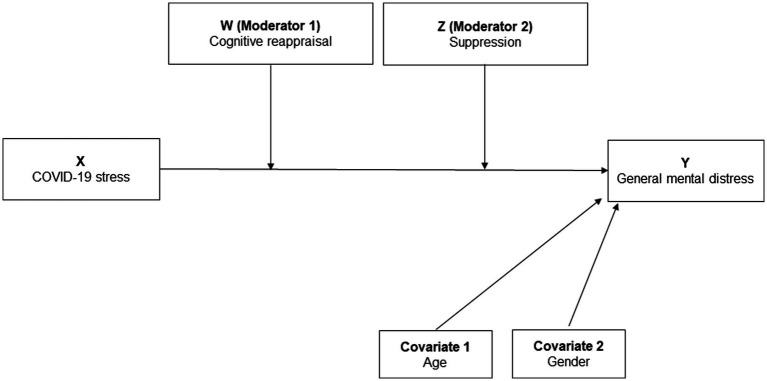
Conceptual model of how cognitive reappraisal (ERQ-CR) and suppression (ERQ-S) moderate the relationship between COVID-19 stress and general mental distress (PHQ 4) (double moderator, PROCESS model 2, *n* = 1,207).

**Figure 2:**
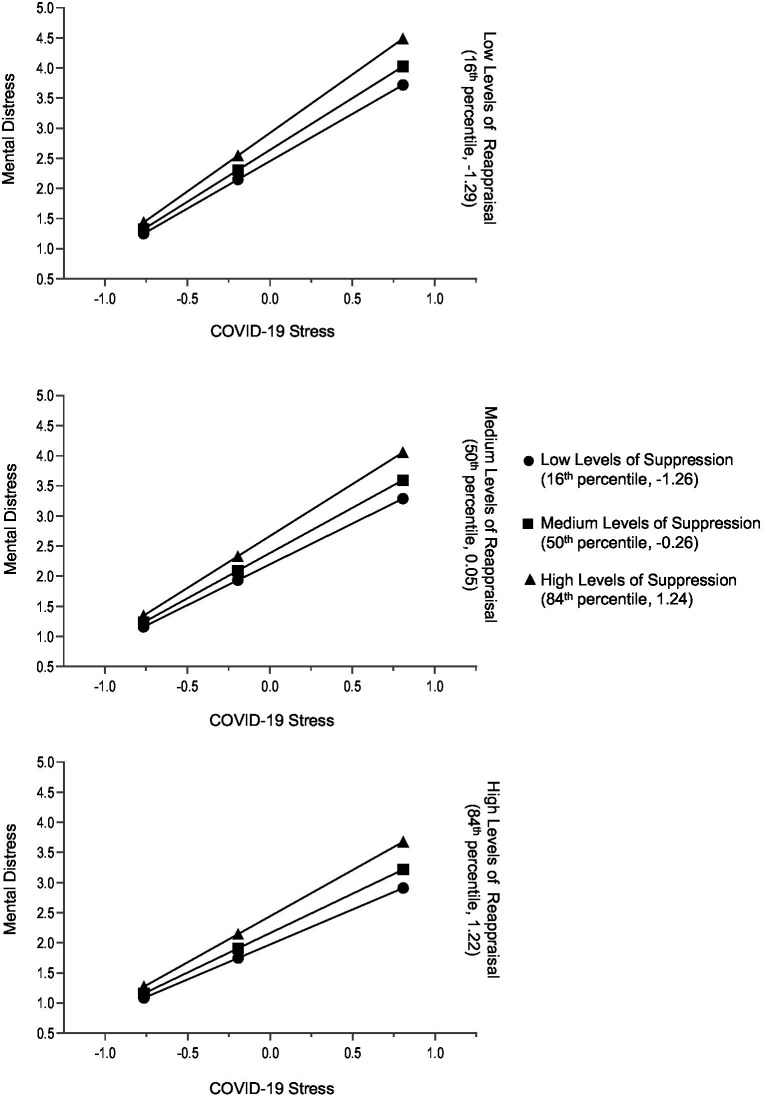
Total sample: conditional effects of COVID-19 stress on general mental distress for low, medium, and high levels of reappraisal and low, medium, and high levels of suppression. The variables ERQ Suppression, ERQ Reappraisal, and COVID-19 Stress Scale were mean centered prior to analysis (*n* = 1,207).

The aims of this study were to (1) investigate whether emotion regulation moderated the relationship between COVID-19 stress and general mental distress during the first months of the pandemic in a community sample from Norway (Figure 1), and (2) examine in an exploratory way to what extent there were gender differences in the interrelations between COVID-19 stress, emotion regulation, and mental distress. After descriptive analyses, we performed moderation analyses for the complete sample. In the next step, we compared men and women regarding the study variables and repeated moderation analysis separately for women and men. We argue that high cognitive reappraisal will buffer the relationship between COVID-19 stress and general mental distress, and suppression will strengthen the relationship between COVID 19 stress and general mental distress. Previous research, using the same dataset, showed that COVID-19 stress was positively related to general mental distress in a population in Norway during the early months of the COVID-19 pandemic (Krampe et al., 2021). Consequently, COVID-19 stress and general mental distress are key research variables in this study. Age was included as a covariate, given that research shows emotion regulation behaviour is linked to lifespan (Carstensen et al., 2011). Regarding gender differences, we expected women to show higher scores of cognitive reappraisal and men to show higher scores of expressive suppression. Given the conflicting evidence on gender effects within the relations of stress, emotion regulation and mental health, the separate moderation analyses for women and men were performed in an explorative way.

## Materials and methods

### Sample and procedures

This cross-sectional study was conducted in Norway (*n* = 1,225) from the period May 26th to June 4th 2020. During this time the Norwegian Government’s COVID-19 regulations were gradually eased (Hansen et al., 2021). The study was distributed on several web pages of the collaborating institutions (e.g., Innlandet Hospital Trust, MF Specialized University, University of Stavanger) as well as forwarded by social media (e.g., Facebook). All participants expressed their informed consent by explicitly agreeing to continue with the questionnaire after being informed about the study’s aims, employed data protection, participants’ rights, and contact points for questions or concerns. Participation was voluntary and with no compensation. Ethical approval was obtained by Personvernombudet Innlandet Hospital Trust, Norway, No. 20/02104–1.

### Measures

#### COVID-19 stress

The COVID-19 Stress Scale comprises seven items and involves a broad range of affective stress reactions to the pandemic situation (e.g., feelings of intolerability, boredom, anger and being left alone) as well as fears and pessimism about personal resources and the future (Schnell and Krampe, 2020). Sample items include *“I am irritated” and “I am afraid of the pandemic and what will happen to us.”* The response scale ranged from 0 (*strongly disagree*) to 5 (*strongly agree*). Cronbach’s alpha in this study was α = 0.73. Confirmatory factor analyses were conducted showing a one-dimensional model of COVID-19 stress (Krampe et al., 2021). The scale has proven valid in several studies (Schnell and Krampe, 2020, 2022a,b; Dyrendal and Hestad, 2021; Krampe et al., 2021; Schnell et al., 2021).

#### General mental distress

General mental distress was measured with the Patient Health Questionnaire 4, PHQ-4 (4 items) (Kroenke et al., 2009, 2010). The PHQ-4 has demonstrated good reliability and validity in both clinical and population samples in Norway (Andreassen et al., 2019; Solem et al., 2021) and measures core symptoms of current depression and anxiety. Participants were asked to respond to the items in regard to the past two weeks (*“Over the last 2 weeks, how often have you been bothered by the following problems?”*). Sample items included “*Little interest or pleasure in doing things”* (depression) and “*Feeling nervous, anxious or on edge”* (anxiety). The response scale ranged from 0 = not at all to 3 = nearly every day. The sum score ranges from 0 to12, and cut-off points > 2, >3, and > 5 indicate mild, moderate, and severe mental distress, respectively (Kroenke et al., 2009; Kerper et al., 2014). Cronbach’s alpha in this study was *α* = 0.82.

#### Emotion regulation

Emotion regulation was measured using the 10-item Emotion Regulation Questionnaire (ERQ: Gross and John, 2003). This is one of the most widely used instruments to measure emotion regulation and has shown good psychometric properties in both clinical and population samples (Gómez-Ortiz et al., 2016; Sætren et al., 2019; Preece et al., 2020). Instructions were adjusted to how participants generally regulated their emotions when encountering stressful situations during COVID-19 restrictions. Participants were asked to rate the degree to which they regulated their emotions *via* cognitive reappraisal (6 items, sample item, e.g., “*When I want to experience more positive emotions, I change my way of thinking”*) – or *via* suppression (4 items, *sample item,* e.g.*, “I control my feelings by not expressing them”*). The response scale ranged from 1 (*strongly disagree*) to 7 (*strongly agree*). The measures were translated from English into Norwegian and then professionally and independently back-translated to ensure language equivalence. After some adjustment, the back-translation was approved by the original developer James Gross (personal communication). In this study, Cronbach’s alphas of the two subscales were α = 0.88 for cognitive reappraisal and α = 0.81 for suppression.

### Statistical analyses

For all statistical tests, a two tailed value of *p* < 0.05 was considered statistically significant. Due to their small number, data from participants identifying as gender diverse were excluded from analyses that contained gender as a variable (*n* = 2). To test whether cognitive reappraisal and suppression moderate the relationship between COVID-19 stress and general mental distress (HQ1) a double moderation analysis was conducted using PROCESS 4.1 macro for SPSS (version 27), model 2 (Hayes and Andrew, 2013; Figure 1). PROCESS is a widely used regression-based path analytic approach to modeling mediation and moderation relationships, and therefore appropriate for testing our first hypothesis (Hayes et al., 2017). The moderation analyses employed bootstrapping with 5,000 samples. Independent *t*-test and a gender specific moderation analysis were applied to evaluate any gender differences in emotion regulation.

## Results

### Sample characteristics and correlational analyses

Means, standard deviations and inter-correlations among the study variables are reported in Table 1. While COVID-19 stress and general mental distress showed a large positive correlation, all other correlations were of moderate to small size. Both COVID-19 stress and general mental distress correlated negatively with cognitive reappraisal and positively with suppression. Younger age was related to higher values in COVID-19 stress, general mental distress, and suppression. Gender was only marginally related to COVID-19 stress, general mental distress, cognitive reappraisal and age, but markedly associated with suppression (Table 2).

**Table 1:** Sociodemographic characteristics of the sample (*N* = 1,225).

	Number or mean	% or SD
Age (Years)^+^	50.27	13.16
Gender Women Men Diverse	897 326 2	73.2 26.6 0.2
Nationality Norwegian Denmark Sweden Other nationalities	1,170 8 17 30	95.5 0.7 1.4 2.4
University education	1,073	87.6
Occupation Health care^a^ Education system^b^ Different industries^c^ Currently not working	325 238 482 180	26.5 19.4 39.4 14.7

^+^*n* = 1,209. ^a^e.g., nurses, doctors, psychologists. ^b^e.g., kindergarten, school, higher education. ^c^e.g., cultural sector, social organizations, hotel and catering industry, trade, and police.

**Table 2:** Descriptive statistics, reliabilities and inter-correlations among variables^+^.

	*M*	SD	1	2	3	4	5
COVID-19 stress	1.34	0.82	**0.73**				
General mental distress	2.51	2.35	0.61^**^	**0.82**			
Cognitive reappraisal	4.45	1.27	−0.20^**^	−0.21^**^	**0.88**		
Suppression	2.51	1.20	0.19^**^	0.23^**^	−0.04	**0.81**	
Age	50.27	13.16	−0.12^**^	−0.19^**^	0.01	−0.10^**^	
Gender			0.03	0.05^*^	0.06^*^	−0.17^**^	−0.06^*^

^+^Reliability estimates are shown in bold along the diagonal; *n* = 1,207–1,225, due to missing data; Measures: COVID-19 stress: COVID-19 Stress Scale (range: 0–5); general mental distress: PHQ-4 (range: 0–12); emotion regulation (cognitive reappraisal, suppression): ERQ (range: 1–7); Gender: 1 = male (*n* = 326), 2 = female (*n* = 897). ^*^p < 0.05, ^**^p < 0.001, two-tailed.

### Moderating role of cognitive reappraisal and suppression

Cognitive reappraisal and suppression moderated the relationship between COVID-19 stress and general mental distress (Table 3). As expected, the positive relationship between COVID-19 stress and general mental distress was moderated negatively by cognitive reappraisal (*B* = −0.17, *SE* = 0.05, *p* ≤ 0.001) and positively by suppression (*B* = 0.15, *SE* = 0.05, *p* = 0.002) (Table 3). Both cognitive reappraisal and suppression acted as independent moderators (Figure 2). When COVID-19 stress was low, general mental distress was also low, regardless of the extent of emotion regulation through cognitive reappraisal or suppression. However, with increasing COVID-19 stress, general mental distress levels were significantly higher for those who suppressed their emotions and for those who used less cognitive reappraisal. The model was significant at *F*(7, 1,199) =125.22, *p* < 0.001. COVID-19 stress, cognitive reappraisal, suppression, age and gender explained 42% of the variance in general mental distress.

**Table 3:** Cognitive reappraisal and suppression as moderators of the relationship between COVID-19 stress and general mental distress; *n* = 1,207.

PHQ-4			95% CI for estimate		
	*B* (SE)	*t*	LL	UL	*p*
Intercept	2.86 (0.30)	9.50	2.27	3.45	<0.001
COVID-19 Stress (IV)	1.55 (0.07)	23.07	1.42	1.68	<0.001
Reappraisal (Mod. 1)	−0.19 (0.04)	−4.59	−0.27	−0.11	<0.001
Interaction IV × Mod1	**−0.17 (0.05)**	**−3.28**	**−0.26**	**−0.07**	0.001
Suppression (Mod. 2)	0.19 (0.05)	4.19	0.10	0.28	<0.001
Interaction IV × Mod2	**0.15 (0.05)**	**3.06**	**0.05**	**0.24**	0.002
Gender	0.29 (0.12)	2.47	0.06	0.53	0.014
Age	−0.02 (0.01)	−4.64	−0.03	−0.01	<0.001

Measures: COVID-19 stress: COVID-19 Stress Scale (range: 0–5); general mental distress: PHQ-4 (range: 0–12); emotion regulation (cognitive reappraisal, suppression): ERQ (range: 1–7); Gender: 1 = male, 2 = female. Bold values refer to the interaction effects.

### Comparisons of women and men

There were no statistically significant differences between women and men concerning mean scores of COVID-19 stress, general mental distress, or age (Table 4). Moreover, women and men did not differ significantly regarding rates of general mental distress for the cut-offs >5 (w: 9.3%, m: 8.3%, *p* = 0.600), >3 (w: 30.4%, m: 24.8%, *p* = 0.057), and >2 (46.6%, m, 40.5% *p* = 0.058). Consistent with our hypotheses, men reported using suppression to a higher degree than women, with a small to medium observed effect (Hedges’ *g* = 0.4). Women, on the other hand, used significantly more reappraisal than men, with a rather small observed effect (Hedges’ *g* = −0.15).

**Table 4:** Comparison of women and men regarding study variables.

	Women (*n* = 897)	Men (*n* = 326)				95% CI	
	*M* (SD)	*M* (SD)	*t*	*p*	Hedges’ *g*	Lower	Upper
COVID-19 stress	1.35 (0.83)	1.30 (0.77)	−0.91	0.364	−0.06	−0.19	0.07
General mental distress	2.57 (2.32)	2.29 (2.37)	−1.87	0.061	−0.12	−0.25	0.01
Reappraisal	4.50 (1.24)	4.32 (1.32)	−2.25	0.025	−0.15	−0.27	−0.02
Suppression	2.38 (1.15)	2.85 (1.28)	5.85	< 0.001	0.40	0.27	0.53
Age^+^	49.79 (12.52)	51.60 (14.72)	1.96	0.050	0.14	0.01	0.27

^+^n = 1,207, due to missing data. Measures: COVID-19 stress: COVID-19 Stress Scale (range: 0–5); general mental distress: PHQ-4 (range: 0–12); emotion regulation (cognitive reappraisal, suppression): ERQ (range: 1–7). Gender: 1 = male, 2 = female.

**Figure 3:**
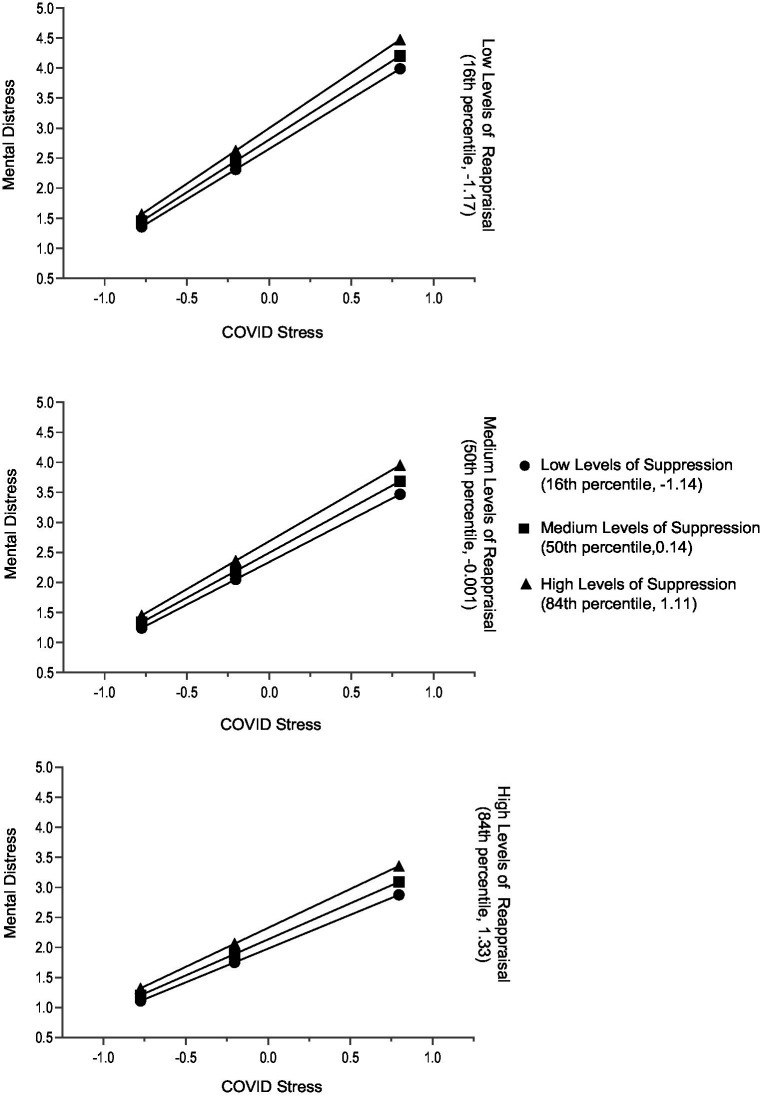
Women: conditional effects of COVID-19 stress on general mental distress for low, medium, and high levels of reappraisal and low, medium, and high levels of suppression. The variables ERQ Suppression, ERQ Reappraisal, and COVID-19 Stress Scale were mean centered prior to analysis (*n* = 884).

Separate moderation analyses were performed for women and men. Both regression models were significant: For women at *F*(6, 877) = 106.82, *p* < 0.001, and 42% of the variance explanation in general mental distress; for men at *F*(6, 316) = 142.54, *p* < 0.001, and 45% of the variance explanation in general mental distress. The models revealed significant gender differences. In women, the directions of regression coefficients remained equal to the ones of the regression model for the total sample. However, the effect sizes were higher for reappraisal and its interaction with COVID-19 stress and slightly lower for suppression. Importantly, the interaction of suppression and COVID-19 stress was not significant (Table 4). Independent of each other, COVID-19 stress and suppression were associated with higher, and reappraisal with lower mental distress. With increasing COVID-19 stress, mental distress levels were not significantly different for women who used more suppression. However, they were significantly higher for those who used less cognitive reappraisal (Table 5 and Figure 3).

**Table 5:** Differential moderation effects of emotion regulation in women and men: cognitive reappraisal and suppression as moderators of the relationship between COVID-19 stress and general mental distress.

(A) Women (*n* = 884)			95% CI for estimate		
	*B* (SE)	*t*	LL	UL	*p*
Intercept	3.33 (0.25)	13.46	2.84	3.81	<0.001
COVID-19 Stress (IV)	1.51 (0.08)	19.58	1.35	1.66	<0.001
Reappraisal (Mod. 1)	−0.27 (0.05)	−5.44	−0.37	−0.17	<0.001
Interaction IV × Mod1	**−0.22 (0.06)**	**−3.77**	**−0.34**	**−0.11**	<0.001
Suppression (Mod. 2)	0.15 (0.05)	2.83	0.05	0.26	0.005
Interaction IV × Mod2	**0.08 (0.06)**	**1.40**	**−0.03**	**0.19**	**0.161**
Age	−0.02 (0.01)	−3.39	−0.03	−0.01	<0.001
(B) Men (*n* = 323)			95% CI for estimate		
	*B* (SE)	t	LL	UL	*p*
Intercept	3.30 (0.38)	8.74	2.55	4.03	<0.001
COVID-19 Stress (IV)	1.65 (0.14)	12.01	1.38	1.91	<0.001
Reappraisal (Mod. 1)	−0.02 (0.08)	−0.25	−0.17	0.13	0.802
Interaction IV xMod1	**−0.03 (0.10)**	**−0.33**	**−0.22**	**0.16**	0.746
Suppression (Mod. 2)	0.26 (0.08)	3.25	0.10	0.42	0.001
Interaction IV × Mod2	**0.38 (0.10)**	**3.64**	**0.17**	**0.58**	<0.001
Age	−0.02 (0.01)	−2.93	−0.03	−0.01	0.004

Measures: COVID-19 stress: COVID-19 Stress Scale (range: 0–5); general mental distress: PHQ-4 (range: 0–12); emotion regulation (cognitive reappraisal, suppression): ERQ (range: 1–7); Gender: 1 = male, 2 = female. Bold values refer to the interaction effects.

**Figure 4:**
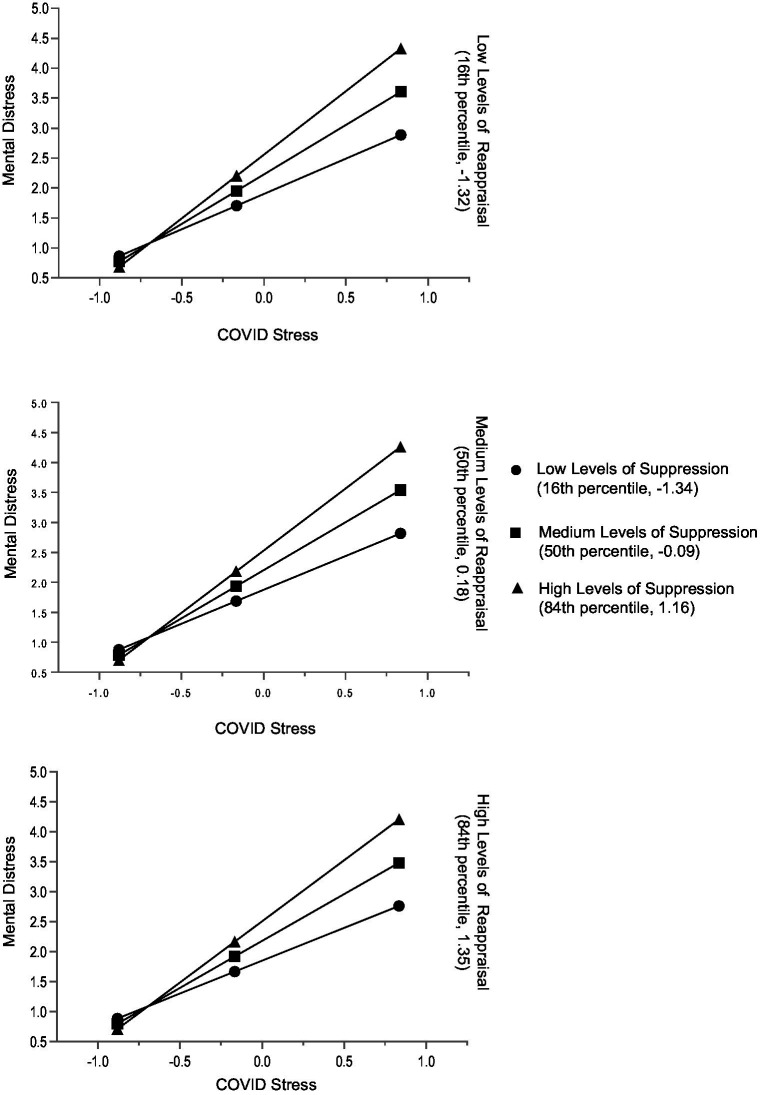
Men: conditional effects of COVID-19 stress on general mental distress for low, medium, and high levels of reappraisal and low, medium, and high levels of suppression. The variables ERQ Suppression, ERQ Reappraisal, and COVID-19 Stress Scale were mean centered prior to analysis (*n* = 323).

In men, both the main regression effects of reappraisal and its interaction effect with COVID-19 stress were not significant. Compared with the model of the total sample, the main effect of suppression and its interaction effect with COVID-19 stress were larger (Table 5). Independent of each other, COVID-19 stress and suppression were associated with higher mental distress. With increasing COVID-19 stress, mental distress levels were significantly higher for men who used more suppression (Table 5 and Figure 4). The results of the gender-specific moderation analyses are also supported by the zero-order correlational patterns of the subsamples of women and men (Supplementary Table S1).

## Discussion

### Major findings

This study investigated whether cognitive reappraisal and expressive suppression moderated the relationship between COVID-19 stress and general mental distress during the early months of the COVID-19 pandemic in Norway. We found that cognitive reappraisal and suppression were differently related to COVID-19 stress and had opposite associations with general distress. Moreover, both variables served as moderators of the relationship between COVID-19 stress and general mental distress. While suppression worsened the relationship between COVID-19 stress and general mental distress, cognitive reappraisal buffered the relationship. When COVID-19 stress was low, this was also the case for general mental distress, and the mode of emotion regulation did not matter. This changed with increasing severity of COVID-19 stress. Here, lower cognitive reappraisal and higher emotion suppression were associated with elevated levels of general mental distress. Our findings revealed two significant gender differences. (1) Men had higher scores of expressive suppression, and women had higher scores of cognitive reappraisal. (2) Gender-specific moderation analyses suggest differential ways of emotion regulation. In women, cognitive reappraisal showed a significant negative relation to mental distress, and a substantial buffering effect of the relation between COVID-19 stress and mental distress. While suppression was positively related to higher distress, it did not buffer the relation between COVID-19 stress and mental distress. In men, cognitive reappraisal was not significantly related to mental distress, and it did not show a buffering effect. However, suppression was significantly associated with higher mental distress, and as a moderator, it substantially exacerbated the association of COVID-19 stress and mental distress. All in all, our data suggest that constructive emotion regulation became particularly relevant in situations of high COVID-19 stress.

Overall, our results are consistent with pre-pandemic research on emotion regulation showing that reappraisal buffered and suppression exacerbated associations between stress and symptoms of mental distress like anxiety and depression (e.g., Troy et al., 2010; Vanderhasselt et al., 2014; Boyes et al., 2016). The findings of the study at hand are also in line with recent COVID-19 studies that replicated the stress-distress buffering effect of cognitive reappraisal and the exacerbating effect of expressive suppression (Prikhidko et al., 2020; Xu et al., 2020; Yang et al., 2020; Gröndal et al., 2021; Kuhlman et al., 2021; Raio et al., 2021; Ye et al., 2021; Zhang et al., 2021; Chen et al., 2022; Vertsberger et al., 2022).

Our results concerning gender-specific differentiations confirm extensive previous research that suggested gender differences in emotion regulation (Tamres et al., 2002; Nolen-Hoeksema, 2012; Goubet and Chrysikou, 2019; Rogier et al., 2019). The higher use of reappraisal by women and of suppression by men is in accordance with several previous studies before and during the COVID-19 pandemic (Tamres et al., 2002; Flynn et al., 2010; Nolen-Hoeksema and Aldao, 2011; Megıas-Robles et al., 2019; Rogier et al., 2019; Canlı and Karaşar, 2020; Santi et al., 2021; Li et al., 2022).

We are unaware of previous investigations comparing women and men regarding stress-distress buffering and exacerbating moderator effects of cognitive reappraisal and expressive suppression, respectively. However, our data fit into the puzzle of different gender-specific results of moderation analyses of emotion regulation before the COVID-19 pandemic (Flynn et al., 2010; Nolen-Hoeksema, 2012; Megıas-Robles et al., 2019; Rogier et al., 2019; Zhang et al., 2020; Jiang et al., 2022; Lutz et al., 2022), and also during the pandemic (Muñoz-Navarro et al., 2021; Santi et al., 2021; Panno et al., 2022; Rodas et al., 2022). At this point, we suggest that further studies are necessary with the aim of exploring potential superordinate factors and processes to explain common patterns and pathways underlying the diverse findings on specific moderation effects.

#### The role of cognitive reappraisal and suppression in stressful COVID-19 times

As previously stated, people’s emotion regulation abilities seem to be influenced by contextual factors (Troy et al., 2010; Gross, 2015; Tamir, 2016; Troy et al., 2017). Research shows that in contexts of uncontrollable stressors, cognitive reappraisal becomes particularly beneficial for people’s mental health. However, contextual factors are defined as all intervening factors that affect a complex phenomenon (c.f. Ploeg et al., 2019) (e.g., transactions between individuals and the environment) (Nolen-Hoeksema, 2012). Examples of (external) contextual factors in this study are the culture, welfare, state of health, living conditions, severity of the COVID-19 restrictions, COVID-19 mortality rates, leadership (authorities) and political trust. The Norwegian lockdown was relatively short in duration (From March 11th 2020, with a gradual relaxation in April 2020). At the time of performing this study, the authorities in Norway were gradually dispensing with some of COVID-19 restrictions (Ursin et al., 2020), although social distancing, social isolation, working from home and travel restrictions were still applicable (Knudsen et al., 2021). Health and welfare are contextual factors that may have influenced our findings (Sachs et al., 2022). Norway is ranked among the top 10 countries of the world in GNP *per capita*, with a high level of welfare and strong and resilient national health systems, including public health (OECD, 2020; Britannica, 2022), (e.g., Sachs et al., 2022). The COVID-19 mortality rate in Norway was low (0.45%) compared to its neighboring countries (1.6–2%) and further afield, such as Italy and Spain (7–10%) (Sciencenorway, 2022). Another contextual factor that may have influenced our findings was that the public had a high level of trust in the Norwegian authorities and viewed them as being transparent and honest (Offerdal et al., 2021; Ihlen et al., 2022).Moreover, lifespan (individual factors) also appears to influence people’s emotion regulation behaviour (Carstensen et al., 2011; Kunzmann and Wrosch, 2018). Lifespan changes are associated with improved emotional stability (e.g., using more adaptive emotion regulation strategies) and well-being (Martins et al., 2016; Livingstone and Isaacowitz, 2018). A comparative study of COVID-19 stress and general mental distress between Norway and Germany/Austria (Krampe et al., 2021) (same data set) shows that Norwegian citizens had better mental health, indicated by less COVID-19 stress and general mental distress. Other studies comparing mental health/well-being before and during the onset of the pandemic in Norway found only a slight increase in mental health issues, particularly in vulnerable groups (Hoffart et al., 2020; Ebrahimi et al., 2021). However, constructive use of cognitive reappraisal is not always easy (Troy et al., 2018) and requires sufficient mental resources, such as vitality and mental well-being (Troy et al., 2018; Haver et al., 2021; Wu et al., 2021). Since cognitive reappraisal is closely linked to lifespan (average age: 52 years) and mental health/well-being, we believe that contextual and individual factors may have influenced our findings.

In terms of suppression, numerous COVID-19 studies indicate that lack of social support, loss of community and friendship worsened psychological distress (e.g., Banerjee and Rai, 2020; Jain et al., 2020; Smith et al., 2020; Philpot et al., 2021; von Mohr et al., 2021). It is well-researched that social connectedness e.g., social networking, community, engagement and friendly touch, acts as a buffer against various forms of distress (for review, see Gariépy et al., 2016). Having positive relationships is associated with mental health, particularly in times of crisis and involves the opportunity to express both negative and positive emotions through verbal and non-verbal communication (Phutela, 2015). It may thus be argued that worries, together with social distancing and social isolation (e.g., felt left to themselves), may have diminished the likelihood of using cognitive reappraisal or other adaptive emotion regulation strategies. Suppressors often experience lack of communication skills and poor relationships and they do not normally share their emotional experiences with others (Gross, 2015). In the long term this may lead to rumination thinking or at worst catastrophic thinking (Aldao and Nolen-Hoeksema, 2010), undermining psychological health (Aldao et al., 2010). That said, we do not yet know whether suppression was frequently or chronically used by the study subjects before the pandemic. Thus, this interpretation has to be handled with caution. Finally, we found a small to medium negative association between age and suppression, showing that younger people are more prone to using suppression. Most research shows that older adults are more consistent in their emotion regulation pattern across situations and are more likely to regulate their emotions by engaging in cognitive reappraisal (John and Gross, 2004; Webb et al., 2012; Sims et al., 2015). These differences in emotion regulation may be linked to the fact that older adults have longer life experience: they are less physiologically reactive, they experience higher well-being and they have learned which strategies are most effective to achieve their personal goals (Carstensen et al., 2011; Eldesouky and English, 2018).

### Gender differences in emotion regulation

Previous research shows gender differences in emotion regulation, particularly in terms of a flexible use of emotion regulation (Nolen-Hoeksema and Aldao, 2011; Goubet and Chrysikou, 2019). Flexible use of emotion regulation (e.g., contextual emotion regulation) refers to matching emotion regulation strategies to environmental circumstances (Aldao et al., 2015; Ullah et al., 2018). Our findings revealed that cognitive reappraisal was negatively related to general mental distress in women but not in men. The moderating role of cognitive reappraisal was significant for women but not for men. This may be linked to previous research arguing that women are more interpersonally oriented – they are more likely than men to seek social support in stressful times and are more prone to experiencing and expressing emotions. Women also have a larger repertoire of different emotion regulation strategies and are more skilled in the emotional domain than men (Brackett et al., 2006; Nolen-Hoeksema, 2012; Chaplin and Aldao, 2013). Several investigations have revealed that women are better at cognitive reappraisal (Tamres et al., 2002; Nolen-Hoeksema and Aldao, 2011; Megıas-Robles et al., 2019; Rogier et al., 2019; Li et al., 2022). These studies, along with our data, may indicate that men engage less in cognitive reappraisal but it is also possible that men use cognitive reappraisal unconsciously and thus do not report it (Nolen-Hoeksema, 2012).

In terms of suppression, our findings revealed that suppression was positively related to general mental distress in both women and men. When examining the moderation role, suppression was significant for men but not for women. These gender differences can be linked to the flexible use of emotion regulation, which involves a sensitivity to the situational demands (context), ability to use different emotion regulation strategies (large repertoire) and the ability to switch emotion regulation strategy if needed (Aldao et al., 2015; Eldesouky and English, 2018; Goubet and Chrysikou, 2019). Notably, flexible use of emotion regulation is found to be adaptive when it results in an enhanced likelihood of achieving personal and meaningful goals (Aldao et al., 2015; Rogier et al., 2019). For example, by engaging in risk-reducing behaviour or responding to environmental demands (e.g., following the government restrictions) the women in this study may have used suppression as a problem-focused strategy, enabling the threat to be endured and/or minimized. Thus, they were able to replace an uncomfortable emotion (anger, fear) with a more acceptable one or protect their relatives from discomfort or pain (e.g., alleviate their relatives’ fear). Suppression may therefore have had a functional and adaptive role during the pandemic, serving to mobilize energy for these women and help them pursue their goals (Tamir, 2016; Rogier et al., 2019). This use of suppression may have enabled women to adapt to the pandemic more effectively (e.g., Troy et al., 2013). Concerning men and their use of suppression, our findings are in line with previous meta-analyses, reviews and recent studies showing that men are more likely to engage in suppressing than women (Aldao et al., 2010; Flynn et al., 2010; Webb et al., 2012; Megıas-Robles et al., 2019; Rogier et al., 2019). These findings were also confirmed during the pandemic (e.g., Italy, Turkey; China) (Canlı and Karaşar, 2020; Santi et al., 2021; Li et al., 2022). A potential explanation is complex but may be linked to women reporting more symptoms of anxiety and depression, for example, through emotional expression, venting of emotions and social support, which in turn decreases suppression. Men on the other hand are more likely to hide, remove, reduce or deflect their emotions and depression symptoms. Men also have a greater tendency to use alcohol to cope (Nolen-Hoeksema, 2012; Cavanagh et al., 2016). However, our findings must be interpreted with caution since gender differences in emotion regulation are relatively complex. The gender difference in emotion regulation can be linked to several factors, such as social cultural norms (e.g., masculinity, femininity), values, personality, stereotypes, emotion regulation abilities (flexibility), and biological and psychological explanations (Matsumoto et al., 2008; Chaplin and Aldao, 2013; Lopez-Zafra and Gartzia, 2014; Costa et al., 2017; Rogier et al., 2019).

### Limitations, future directions, and conclusions

There are several limitations to the present study. Firstly, the study was conducted in Norway with a relatively high level of health and subjective well-being. Surveys conducted to measure life satisfaction in terms of ‘the happiest country’ have ranked Norway in the top 10 since 2012 (OECD, 2020). Cultural beliefs (e.g., collectivistic vs. individual cultures) and cultural differences, such as norms, values, stereotypes, are all important moderators and mediators in the relationship between emotion regulation and psychological health (Hu et al., 2014; Troy et al., 2018). Moreover, contextual factors, such as welfare, trust in government, infection rates and morality rates during the outbreak of the pandemic are also important aspects in any choice of emotion regulation. Investigating emotion regulation in different countries and contexts is thus required. Secondly, although the study covered a wide age range, the sample was not representative of the general population. Gender differences, as well as education level and life span may have influenced choice of emotion regulation. The sample may thus have been biased, with an overrepresentation of women, relatively high average age and highly educated individuals. Thirdly, this study has surveyed cognitive reappraisal and suppression, two of many identified strategies that individuals use to regulate their emotions (Parkinson and Totterdell, 1999). In order to further increase our understanding of which emotion regulation strategies constitute effective emotion regulation during crisis, a wider range of strategies should be included (e.g., maladaptive emotion regulation: rumination, catastrophizing). Fourthly, to strengthen the validity and to extend our knowledge during (global) crisis, longitudinal studies are required to see how individuals’ emotion regulation strategies fluctuate over time and the extent of their impact on psychological health, including short-and long-time effects of emotion regulation. Finally, to expand our conceptual understanding of emotion regulation during crises and to generate relevant hypotheses future research should include more qualitative studies. Despite these limitations, the convenience sampling of this material is coherent and consistent. The present study is well suited as a study aiming to explore new knowledge about COVID-19 stress, emotion regulation and general mental distress during a global health crisis.

This study examines emotion regulation in times of crises. The study also provides a more nuanced understanding of the role of cognitive reappraisal and suppression in a Scandinavian context. Even though COVID-19 now appears to be under control in most countries, it is important to note that new variants of the virus and new pandemics, alongside energy crises, economic crises and natural disasters may arise in the future. Given that cognitive reappraisal and suppression have different influences on the relationships between COVID-19 stress and general mental distress, health authorities should implement emotion regulation programs to promote a healthy and flexible use of emotion regulation. In practical terms, our findings suggest preventive measure responses through health promoting programs for developing/building psychological resilience in adolescents throughout their lives. Vulnerable groups, such as the youngest, would particularly benefit from emotion regulation training in times of crisis (e.g., web-based mindfulness programs). Health authorities should therefore be encouraged to develop health promoting programs (e.g., online self-guided), followed by preventive measures and interventions in school. Moreover, the findings in this study emphasize the importance of considering gender differences in future emotion regulation research. Consequently, it is vital to acknowledge that differences may exist between genders in terms of emotion regulation. This requires gender awareness, e.g., tailored programs for men and women.

## Data availability statement

The raw data supporting the conclusions of this article will be made available by the authors, without undue reservation.

## Ethics statement

The studies involving human participants were reviewed and approved by Personvernombudet Innlandet Hospital Trust, Norway, No 20/02104–1. All participants expressed their informed consent by explicitly agreeing to continue with the questionnaire after being informed about the study’s aims, employed data protection, participants’ rights, and contact points for questions or concerns. All methods were carried out in accordance with relevant guidelines and regulations. Written informed consent for participation was not required for this study in accordance with the national legislation and the institutional requirements.

## Author contributions

AH wrote the first draft of the manuscript and, together with HK, TS, and LD performed the statistical analyses. LD and GS conceptualized, developed, and concluded the survey. AH contributed to the conceptualization and development of the survey. LD, TS, and HK performed data curation and project administration of the data set. AH, HK, and TS finalized the manuscript. All authors discussed the results, commented on the manuscript, contributed to critical revision of the manuscript, and read and approved the final manuscript.

## Funding

This study was funded by Innlandet Hospital Trust. Research number/Grant: 677300.

## Conflict of interest

The authors declare that the research was conducted in the absence of any commercial or financial relationships that could be construed as a potential conflict of interest.

## Publisher’s note

All claims expressed in this article are solely those of the authors and do not necessarily represent those of their affiliated organizations, or those of the publisher, the editors and the reviewers. Any product that may be evaluated in this article, or claim that may be made by its manufacturer, is not guaranteed or endorsed by the publisher.
