# Supplementary material for: Emotion regulation moderates the association between COVID-19 stress and mental distress: findings on buffering, exacerbation, and gender differences in a cross-sectional study from Norway
Source: Front Psychol. 2023 Jun 22;14:1121986. doi: 10.3389/fpsyg.2023.1121986 (PMC10325689; doi:10.3389/fpsyg.2023.1121986)
Supplement: Supplementary file 1 [file Table_1.pdf]

Supplemental table 1. Descriptive Statistics, reliabilities and inter-correlations among variables in women (n=897) and men (n=326) [+]

	**1**	**2**	**3**	**4**	**5**
COVID-19 stress		*.61***	*-.15**	*.16**	*-.22***
General mental distress	.61**		*-.09*	*.28***	*-.28***
Cognitive reappraisal	-.22**	-.26**		*-0.1*	*-.01*
Suppression	.21**	.22**	-.04		-.21**
Age	-.08**	-.15**	.01	-.10**	

[+] Data of women in the lower left part, data of men in italics in the upper right part of the table.
Measures: COVID-19 stress: COVID-19 Stress Scale (range: 0-5); general mental distress: PHQ-4 (range: 0-12); emotion regulation (cognitive reappraisal, suppression): ERQ (range: 1-7);
$^{*}p < .05$, $^{**}p < .001$, two-tailed.
